# Transoral robotic surgery in the management of head and neck tumours

**DOI:** 10.3332/ecancer.2013.359

**Published:** 2013-09-26

**Authors:** Vittorio Rinaldi, Davide Pagani, Sara Torretta, Lorenzo Pignataro

**Affiliations:** Department of Specialist Surgical Sciences, University of Milan, Otolaryngology Department, Fondazione IRCCS Cà Granda Ospedale Maggiore Policlinico, 20122 Milan, Italy

**Keywords:** robotic surgery, head and neck surgery, head and neck tumours, transoral surgery

## Abstract

The article reviews the use of robotic technology for head and neck tumours. The authors discuss the development of transoral robotic surgery (TORS), the current status of the technology, and the set-up in the operating room. The article provides a review of the literature, highlighting the applications, advantages, functional outcomes, and disadvantages of TORS for each anatomic subsite (oropharynx, hypopharynx, larynx, parapharyngeal space, and skull base). New challenges related to reconstruction are also presented. Overall early functional and oncologic outcome data are promising; further long-term prospective trials are still needed to confirm the oncological safety of TORS.

## Introduction

Robotic-assisted surgery has its roots in 1972, when the National Aeronautics and Space Administration began to investigate a method to provide surgical care to orbiting astronauts through telepresence surgery [1].

After the first laparoscopic splenectomy performed in 1997 [2], robotic-assisted surgery has gained popularity in several surgical specialties such as general, urological, cardiothoracic, gynecological, orthopedic, and neurological surgeries [3].

Transoral robotic surgery (TORS) was first introduced in [4] by Weinstein *et al* with a case report of a supraglottic laryngectomy in a canine model and by MacLeod and Melder [5], who reported the excision of a vallecular cyst in a human patient with a setup time of 75 min and a surgical time of 30 min. Since these early reports, the development of TORS has been steadily progressing and many other studies on TORS in animal cadavers, human subjects, and various head and neck cancer sites have been published.

In 2006 and 2007, preclinical and clinical studies by a team at the University of Pennsylvania demonstrated [6–8] the feasibility and safety of transoral resections with the assistance of the Da Vinci Surgical Robot (Intuitive Surgical Inc., Sunnyvale, California, United States), such that the US Food and Drug Administration approved TORS for selected benign and malignant head and neck tumours in December 2009 [9].

Because of the possibility of obtaining superior visualisation and complete resection of tumours with wide margins, TORS seems to represent an alternative to open or endoscopic/microscopic approaches in oral and pharyngolaryngeal oncology—particularly with 5-mm instruments—that allows improved vision, greater ease of use, and a shorter operating time [10].

## Current robotic system

The Da Vinci Surgical System consists of three components: a surgeon’s console, a patient-side robotic cart equipped with four arms, and a high-definition three-dimensional vision cart. Articulating surgical instruments are mounted on the robotic arms, which are introduced into the upper aerodigestive tract through the mouth of the patient and manipulated remotely with master robot manipulators from the surgeon’s console.

As far as head and neck surgery is concerned, usually only three of the four arms are employed: one to handle a 12-mm stereoscopic endoscope at an angle of 0° or 30° and the other two equipped with 5-mm endo wrist instruments (Intuitive Surgical Inc.).

Both the endoscope and the robotic instruments are introduced transorally and allow the surgeon to perform procedures equivalent to traditional surgery, with the advantages of enhanced three-dimensional HD visualisation, wide range of motion with seven degrees of freedom, reduction of hand tremors, possibility of navigating around corners through angled scopes, reduction of fatigue, proper hand– eye coordination, and the possibility of telesurgery and teaching opportunities with more favourable learning curves [3, 11–14].

When compared with open surgical approaches, TORS may avoid disfiguring mandibulotomy; it reduces the need for adjuvant radio and/or chemotherapy and for tracheostomy/gastrostomy, improves the return to normal speech and swallowing, and reduces blood loss and postoperative pain [7, 15–17]. Minimal scarring with reduced risk of wound infection, shorter hospital stays, and shorter recovery times may also improve patients’ quality of life (QOL) [18].

TORS may be applied for benign and malignant lesions of the palate, the palatine tonsils, the base of the tongue, the posterior and lateral pharyngeal wall, the parapharyngeal space, the larynx, and the hypopharynx; main contraindications are reduced mouth opening, incomplete lesion visualisation, mandible involvement, tumours involving >50% of the base of the tongue or of the posterior pharyngeal wall, internal carotid artery, or prevertebral fascia involvement [7].

## Surgical setup

TORS is defined as the surgery performed through the oral cavity that uses a minimum of three robotic arms and allows bimanual manipulation of tissues. The surgeon’s cart should be located at the end of the operating room, allowing free space to manoeuver the surgical cart that is placed on the left side of the patient, opposite to the surgeon [19]. The assistant is seated at the head of the patient. The anaesthesia machine and anaesthesiologist are at the patient’s foot. After induction, the endoscopic tower and scrub table are placed on the right; the patient should be placed in a strictly supine position on the operating table [14]. The surgeon places a Crow–Davis mouth gag (suspended to the bed) to gain surgical exposure and three sterilely draped robotic arms are placed in surgical position: the instruments (atraumatic forceps and electrocautery spatula tip) are introduced 30° laterally from the arm supporting the 0° endoscope and placed in the right and left arms of the robot. In laryngeal and pharyngeal surgeries, a Feyh–Kastenbauer (FK) retractor can be used and a flexible aspiration tube for smoke aspiration is introduced into the nasopharynx through one of the nostrils.

All of the authors stress the importance of the correct positioning of the machine, to allow complete exposition of the lesion and free, unobstructed movements of the robotic arms [3, 11–14].

### Oropharynx

**Tongue base**

In 2006, O’Malley *et al* reported a prospective clinical trial evaluating the use of TORS in three patients with tongue base cancer. They reported an excellent visualisation and tumuor removal possibility. The use of the FK retractor is described to be essential for proper exposure and for instrument manoeuverability. Complete resection with negative surgical margins was achieved in all three cases with an excellent control of bleeding; no intraoperative or postoperative complications were encountered [15].

The use of Da Vinci robot has also been reported for tongue base biopsy in the evaluation of unknown primary tumours of the head and neck [20]. Metha *et al* reported a retrospective study on ten patients with unknown primary tumours of the head and neck. All patients underwent a cervical biopsy, positron-emission tomography/computed tomography, formal endoscopy, bilateral tonsillectomy, and transoral robotic base of tongue resection. Robot-assisted base of tongue resection identified primary tumours in 90% patients with minimal morbidity [21].

Reconstruction should be considered particularly for large defects, to maintain a balance between restoration of bulk and maintaining a sensate mucosal surface, so as not to impair the pharyngeal phase of swallowing and to avoid aspiration [22]. The radial forearm flap is the most frequently used [23]. For smaller defects, secondary healing and remucosalisation seem to provide adequate function preservation.

**Tonsillar region**

TORS radical tonsillectomy has widened the indications for transoral resection, because TORS allows excellent visualisation in all directions, concurrent tongue base resection, and the possibility of better identifying small and large vessels. In the series of 27 patients presented by Weinstein *et al*, TORS seems to represent a valid alternative to transmandibular approaches with free-flap reconstruction. All the patients underwent TORS radical tonsillectomy for invasive squamous cell carcinoma (SCC) of the tonsillar region, staged neck dissection, and adjuvant therapy. Major tumour-related contraindications for TORS radical tonsillectomy were: unresectability of involved neck nodes, mandibular invasion, tongue base invasion requiring a resection of >50% of the tongue base, pharyngeal wall involvement necessitating resection of >50% of the posterior pharyngeal wall, radiological confirmation of carotid artery involvement, and fixation of tumour to prevertebral fascia. Neck dissection was performed one to three weeks following TORS radical tonsillectomy, to avoid the risk of creating a connection between the pharynx and the neck and to avoid additional laryngopharyngeal swelling that might require a tracheotomy. No free-flap reconstruction was needed and the mucosal defect at the end of surgery was allowed to heal by secondary intention. The mean operative time to perform TORS was 1 h and 43 min including a mean of 9 min for exposure and robotic positioning. Complete resection with negative surgical margins was achieved in 93% of the cases. The surgical complication rate was 19%, most cases resolving without significant sequelae: one mucosal haemorrhage requiring a return to the operating room, one tracheostomy for OSA exacerbation, two cases of trismus, and one of hypernasality requiring an outpatient procedure for correction. No mortality occurred and no cases of pneumonia or fistula were observed. Twenty patients were extubated at the end of the surgical procedure, the remaining six patients remained intubated for laryngopharyngeal swelling, treated with steroids for one to three days and then extubated after an average of 2.7 days postoperatively. One patient required unplanned tracheostomy for his sleep aponea exacerbation. All the patients underwent percutaneous gastrostomy and 96% of them recovered a proper swallowing function at the follow-up [7].

For the majority of the patients, no reconstruction is required if the resection is limited to the tonsillar region; free tissue transfer should be considered in patients predisposed to poor wound healing, or if the carotid artery is exposed in the parapharyngeal space [22]. When the resection involves soft palate tissue, restoring competence of the velopharyngeal sphincter is mandatory; palatal defects involving less than one-quarter can be reconstructed with primary closure or simple approximation to the flap, while more extended defects require soft tissue free flaps, mainly radial forearm, and pharyngeal flaps [24].

### Larynx

Weinstein *et al* reported a prospective human clinical trial evaluating the use of TORS in three cases of supraglottic partial laryngectomy for supraglottic carcinoma. An excellent surgical exposure with complete tumour resection was achieved. No intraoperative or postoperative complications were reported. The median overall operation time was 120 min including 18 min for exposure and robotic positioning. Robotic-assisted supraglottic laryngectomy is reported to be technically feasible and relatively safe, representing an alternative to open approaches and conventional transoral supraglottic partial laryngectomy [8].

Solares and Strome reported three cases of CO_2_ laser robotic-assisted supraglottic laryngectomy. The FK laryngo-pharyngoscope was reported to provide excellent visualisation in only one patient, and the procedure was completed successfully. In the other two cases, adequate exposure could not be achieved and more traditional techniques were performed [25].

Park *et al* [26] reported a prospective human trial with four patients with glottic carcinoma successfully treated with TORS; no perioperative complications were reported and all surgical margins were disease free.

### Hypopharynx

Although hypopharyngeal SCC represents a clinically distinct and less frequent entity among other cancers of the head and neck, early experiences using TORS for hypopharyngectomy for I- and II-staged cancers seem to be promising.

Park *et al* reported a series of ten patients with T1 or T2 pyriform sinus and posterior pharyngeal wall cancer successfully treated with TORS. The mean operation time was 62.4 min with an average of 17.5 min for the robot setup. No significant perioperative complications were reported. A return to normal swallowing function was observed in all patients within 8.3 days average and decannulation within 6.3 days after surgery. The authors concluded that transoral robotic hypopharyngectomy is feasible and safe for the treatment of early hypopharyngeal tumours [27].

### Parapharyngeal space and infratemporal fossa

TORS used in skull base surgery was first assessed in [28] on animal and cadaver models. They also reported one case of benign neoplasm of the parapharyngeal space and infratemporal fossa treated with TORS. Robotic approach allowed an excellent access, visualisation, and tissue dissection within the parapharyngeal space and infratemporal fossa, providing a safe identification of the internal carotid artery and cranial nerves, and excellent haemostasis. However, certain limitations were observed in the TORS approach, notably its inability to perform wide resection, which may be required for invasive malignant neoplasm. Therefore, the authors concluded that TORS may be best suited for excising well-circumscribed benign lesions, adenomas or schwannomas, and possibly limited parapharyngeal metastatic lymph nodes.

In 2010, the same authors assessed the outcomes of ten patients undergoing parapharyngeal space resection using the TORS approach [29]. The surgery was performed in 90% of the cases without significant complications, with acceptable operative time and blood loss; one case was converted to an open transcervical approach due to difficulties found during TORS resection. In 70% of the cases, it was a pleomorphic adenoma and in those patients the reported local control rate was 100%. A decrease of complication rate was noted in TORS when compared with traditional transcervical surgery.

McCool *et al* [30] reported six complete and two partial dissections of the infratemporal fossa carried out in cadaver models using a suprahyoid port, whereas the second arm and 30° camera were placed transorally.

In cadaveric dissections, Hanna *et al* [31] obtained excellent access to the anterior and central skull base, including the cribriform plate, fovea ethmoidalis, medial orbits, planum sphenoidale, sella turcica, suprasellar and parasellar regions, nasopharynx, pterygopalatine fossa, and clivus.

In skull base surgery, robotic techniques can be considered as the natural evolution of traditional endoscopic techniques [32] and their role is evolving with the goal of maximising surgical resections without compromising oncological principles. Kupferman *et al* [33] reported using robotic technology to greatly facilitate the suture-based dural reconstruction of the anterior cranial base in a cadaver model, ensuring a minimal trauma to surrounding critical neurovascular structures.

## Head and neck robotic reconstructive surgery

As the application of TORS for the treatment of head and neck cancer increases, robotically assisted reconstruction, whether using free flaps, local flaps, or primary closure, holds the potential of expanding TORS applications, keeping the aim to provide patients with less morbid procedures. The introduction of vascularised tissue into an oropharyngeal defect can provide improved functional recovery. The flexibility of the robotic arms allows suture placement transorally in areas of decreased visibility using traditional open techniques. Even microvascular anastomosis has proved faster and more effective [34]. In robotic-assisted microvascular anastomosis, the robotic arms are placed almost horizontally, in the plane of the bed, and in direct proximity of the external incision; a third arm serves as a stationary assistant, while Black Diamond Micro Needle drivers (Intuitive Surgical) and 9.0 nylon suture are used for the anastomosis [35].

Several case series demonstrated the clinically applicable use of the robot for reconstruction of head and neck.

Mukhija *et al* [36] described the first two cases of TORS-assisted free flap (radial forearm) reconstruction of orophayrnx and oral cavity: for both cases, the flaps were inset robotically, while the microanastomosis was performed in the standard fashion.

Selber reported robotic-assisted reconstruction in five patients with oropharyngeal tumours: reconstruction included both local and free flaps, depending on the size of the defect, prior radiation, and the presence or absence of a pharyngotomy. The authors reported the first case of robotic anastomosis, with the advantage of tremor elimination, motion scaling up to 5:1, and an enhanced ability to work precisely in confined spaces. There were no oropharyngeal fistulas. Only one postoperative complication was reported; one patient accidentally bit through his pedicle but without resulting flap failure [35]. Genden *et al* [37] reported a series of 31 patients who underwent TORS reconstruction: promising results are presented with 25 cases of reconstruction with local advancement musculomucosal flap and six with a radial forearm free flap. The authors reported that patients who have been treated with radiation and patients with extensive defects needed free-flap reconstruction because the mucosa was often friable and poorly vascularised. Three patients required a transoral marginal mandibulectomy. All the flaps were inserted robotically, but the anastomosis was done in the standard fashion. None of the patients developed a fistula. Ghanem presented a four-patient case series using robotic free-flap reconstruction. Tumour sites ranged from the tonsil to the base of the tongue and the oral tongue. Three patients received radial forearm free flaps, one a vastus lateralis free flap. All the flaps were inset robotically, with manual microvascular anastomosis. No fistulas were reported, but complications consisted of one neck infection and one partial dehiscence at the anterior tonsillar pillar (allowed to heal by secondary intention) [38].

Longfield *et al* performed 20 robot-assisted oropharyngeal reconstructions, including six anterolateral thigh flaps, five radial forearm flaps, two ulnar artery perforator flaps, two FAMM flaps, five buccal or pharyngeal transposition flaps. Robotic microvascular anastomosis was performed in four of these cases. No flap losses were described [34].

Song *et al* reported a case series of five patients who underwent robotic free-flap reconstruction (four radial forearm free flap and one anterolateral thigh free flap) after TORS for oropharynx tumours. In one case, the microanastomosis was performed using the robotic arms. No major complications were encountered, including flap failure, haematoma, or wound dehiscence [39].

Longfield *et al* provided an algorithmic approach to determine the need of reconstruction after robotic excision of head and neck tumours. TORS is not routinely necessary for tumours within the oral cavity, which usually offer a good manual transoral access. Retromolar trigone represents the only exception and, because of its anatomical features, tumours of this region may benefit from TORS resection and reconstruction: in these cases, local flaps are usually sufficient for reconstruction. Tumours of the oropharynx (tonsil, base of the tongue, and soft palate) are an ideal indication for TORS resection: complicated excisions (involving carotid sheath exposure, bone exposure, and so on) with resulting large defect may benefit from microvascular reconstruction. For supraglottic larynx, Longfield *et al* [34] considered that the reconstruction with free flap is often impractical, although in patients with a good oral aperture, it is technically feasible to resect robotically tumours in the supra- and infrahyoid regions.

## Functional outcomes of TORS

Early TORS data support impressive functional outcomes with low rates of gastrostomy dependency, prompt decannulation, and resumption of normal oral intake (Table 1).

Genden *et al* [16] reported the ability to tolerate an oral diet at a mean of 1.4 days after surgery without any patients requiring gastrostomy tubes. Iseli *et al* reported that 83% patients were tolerating an oral diet within 14 days, while 17% required a feeding tube at 12-month follow-up, and 5.6% demonstrated signs or symptoms of aspiration [37]. Moore *et al* reported that 82% of the patients were tolerating oral diet by the first postoperative visit, whereas 17% required a feeding tube, and none required assistance with feeding at one-year follow-up [38]. Hurtuk *et al* reported that 100% of the patients were able to return to oral diet on the day of surgery and 20% of them required feeding tubes mainly for adjuvant therapy [39].

The majority of the authors reported a low rate of tracheotomy for patients undergoing TORS (see Table 1 for details). Moreover, most of those patients were decannulated within two weeks and no patients required tracheotomy tube at one year after surgery [13].

## Oncological outcomes of TORS

The oncological outcomes from TORS are slowly emerging in the literature and they seem promising [13] (Table 2). In the cohort study, on 47 patients with advanced oropharyngeal carcinoma treated with TORS, Weinstein *et al* reported a local recurrence rate of 2%, a regional recurrence rate of 4%, and a distance recurrence rate of 9% at a minimum of 18 months of follow-up. Overall survival rates were 96% at one year and 82% at two years, with a disease-specific survival of 98% at one year and 90% at two years. The disease-free survival was 96% at one year and 79% at two years. Extracapsular extension in the metastatic nodal disease was found to be statistically affecting the overall survival rates, 38% of the patients avoided chemotherapy and, because of the high rate of negative margins, 11% did not receive adjuvant chemoradiotherapy [40].

Cohen *et al* reported the outcome profile of oropharyngeal SCC treated with TORS comparing two different groups of patients: HPV-positive and negative. In the HPV-positive group (37 patients), the authors reported no local or regional recurrence, though two patients had distant recurrences. In the HPV-negative group, there were no local recurrence, one patient had both regional and distant recurrences. Survival rates showed no statistically significant differences between the two groups of patients, with 81% in HPV-positive group and 80% in the HPV-negative group [41].

White *et al* in a prospective case study of 89 patients with head and neck SCC of all stages and subsites treated with TORS, reported at a median follow-up period of 26 months three patients with local recurrence, seven with regional recurrence (two of whom also had distant metastases), and one patient with distant recurrence; seven patients were treated with TORS for recurrent disease. Of the patients who underwent TORS as primary treatment, 63% underwent adjuvant radiotherapy and 48% had chemotherapy either before or after surgery; 8% underwent salvage TORS after primary chemoradiotherapy. At the last follow-up visit, 82 patients had no evidence of disease, one died of the disease, two died of other diseases, and four were alive with disease. The overall two-year recurrence free survival rate was 86.3% [42].

Genden *et al* in a prospective case-control study of 30 patients with head and neck SCC of all stages and subsites treated with TORS and adjuvant therapy, reported 24 patients alive with no recurrent disease at last follow-up visit. Two patients had local recurrence, two developed distant recurrence, and one developed a second primary tumour. The 18-month locoregional control, distant control, disease-free survival, and overall survival reported rates of 91%, 93%, 78%, and 90%, respectively [17].

## QOL after TORS

Even if the most important outcome for cancer patients is overall survival, in patients with head and neck cancer, QOL, and functional outcome may really be affected by psychological impact of loss of function and physical disfigurement. This has led to a shift to not only organ-preservation treatments, but also function-preservation treatments. Frequent problems include difficulty with speech, respiration, and eating [13]. Leonhardt *et al* reported a return to normal swallowing function and diet one year after surgery in 38 patients with oropharyngeal SCC treated with TORS, while the speech domain was significantly reduced even one year after surgery. They reported that patients who had TORS followed by chemoradiation had significantly lower swallowing scores compared with those without [43].

The case control study on 30 patients with head and neck SCC reported by Genden *et al* found that patients treated with TORS had significantly better short-term swallowing and eating ability and diet compared with patients treated with primary chemoradiation. While TORS patients had a return to baseline in all domains (swallowing, eating, and diet) at 12 months, patients who had chemoradiotherapy did not have a return to baseline in the diet domain [17].

In a cohort of 64 patients who underwent TORS for benign and malignant lesions of the head and neck region, Hurtuk *et al* observed the resumption of normal oral intake in all but one patient, with no patients who needed tracheostomy. Although in all of the patients, the self-administered QOL questionnaire underlined a postoperative decreasing in all of the values, at the one-year follow-up the major part of them returned back to high domains, with the exception of eating function and attitude. QOL scores differed significantly among patients who underwent TORS for malignant diseases compared with those who had a robotic resection for benign lesions; similarly, QOL scores were significantly lower for those patients who underwent adjuvant radio- or chemotherapy and in those affected by tonsillar region cancer [18].

The overall hospital stay is reported to be shorter for TORS patients than for those who would have otherwise undergone an open approach. In the case series of Moore *et al*, all the 35 patients were discharged from the hospital within six days [38]; in the study of Boudreaux *et al*, the mean hospital stay reported was 2.6 days [44], whereas in the experience of Weinstein *et al*, it was between five and seven days [15, 45].

## Conclusions

Development of TORS has greatly facilitated the minimally invasive surgical approach for head and neck carcinoma, circumventing many of the technical limitations commonly associated with transoral laser microsurgical techniques, such as line of sight and two-handed surgery.

TORS provides superb three-dimensional visualisation and magnification in all directions, with tremor filtration, greater freedom of instrument movement facilitating delicate dissection, minimally invasive and less morbid access, and the ability of two surgeons to operate within the field. The excellent ability of bleeding control facilitates *en bloc *complete tumour resection. Technical feasibility, efficacy, and safety have been largely demonstrated by many studies. In patients undergoing TORS, shorter operative time and decreased hospital stay have been observed; excellent oncological and functional outcomes have been also reported [43]. Faster return to normal function allows patients to begin adjuvant therapy, if warranted, much sooner than in patients who undergo open surgery [39], affording potential advantages, and benefits over current treatment modalities of head and neck tumours.

Not all the patients are good candidates for TORS because some of them may present some limiting factors, mostly affecting proper surgical exposure, such as trismus, narrow arched mandible, full dentition, and retrognathia [42]. Access to the tumour may also be influenced by the site and the extension of the tumour, and the equipment employable by the surgeon [16].

The bigger limitation of robotic technology seems to be represented by the absence of sensory tactile feedback, which may give significant drawback in a number of situations [12]. The initial cost of approximately US$1.5 million (coupled with an approximately US$100,000 yearly maintenance fee and US$200/case/disposable instrument) is a significant financial investment and it may clearly hinder widespread use. However, the benefits of decreased operative time and decreased length of hospital stay could offset this cost [45]. Operating room setup time and surgical time can be significantly long at the beginning, but all published articles demonstrated a precipitously and significant reduction of those times as the surgeons gained experience with the Da Vinci Surgical System [42] (Table 3).

In terms of the teachability of TORS, training programs have already yielded successes [45]. As for as oncological results, the evaluations of the surgical margins seem to confirm that the Da Vinci Surgical System may provide, with an accurate patient selection, complete tumour resection with preservation of swallowing function in the majority of cases.

In conclusion, TORS has proven to be a safe and feasible treatment for tumours of the upper aerodigestive tract, and its continuing use and development is encouraged; the results of the published articles are still not sufficient to validate the oncological safety of TORS and further long-term prospective trials are still needed to confirm those results.

## Figures and Tables

**Table 1.** Functional outcomes of TORS.

Author	Primary site	Number of patients	Complications rate (%)	Tracheostomy (%)	Gastrostomy Tube (%)
	Palatine tonsil	27			
	Base of tongue	11			
Hurtuk *et al,* 2011	Larynx	4	0	0	17
[42]	Retromolar trigone	1			
	Parapharynx	1			
	Total	44			
	Tonsil	7			
	Base of tongue	3			
Genden *et al,* 2009	Parapharynx	2	0	0	0
[16]	Hypopharynx	4			
	Palate	2			
	Total	18			
	Oropharynx	18			
Hans *et al,* 2011	Larynx	5	4,5	0	9
[49]	Hypopharynx	2			
	Total	25			
	Oropharynx	44			
Vergez *et al,* 2012	Hypopharynx	64	20,6	15	nd
[50]	Larynx	18			
	Total	126			
	Pharynx	2			
Remacle *et al,* 2012	Supraglottic	2	0	0	nd
[51]	Total	4			
Weinstein *et al,* 2007 [7]	Oropharynx	27	14.8	3.7	4
Park *et al,* 2009 [52]	Tonsil	5	0	100	0
	Tonsil	19			
Moore *et al,* 2009	Base of tongue	26	8,9	31,1	17,8
[41]	Total	45			
	Supraglottic	10			
Lawson *et al*, 2011	Pharynx	10	0	0	nd
[19]	Oral cavity	4			
	Total	24			
	Aryepiglottic fold	3			
	Piriform sinus	2			
	Pharynx	5			
Aubry *et al*, 2011	Base of tongue	3	nd	11,8	11,8
[53]	Vallecula	1			
	Epiglottis	2			
	Arytenoid	2			
	Total	17			
Weinstein *et al*, 2010 [43]	Oropharynx	47	12,8	10,6	2,4
	Supraglottic 2	2			
Benazzo *et al*, 2012	Oropharynx	4	16,7	100	0
[54]	Total	6			
Iseli *et al* 2009	Oropharynx	33			
[40]	Larynx	12	31,5	9,3	16,7
	Oral cavity	6			
	Hypopharynx	3			
	Total	54			
Sinclair *et al*, 2011	Tonsil	29			
[55]	Base of tongue	13	0	0	10
	Total	42			

**Table 2.** Oncological outcomes of TORS.

Author	Primary site	Number of patients	Follow up (months)	Overall survival (%)	Free of disease (%)
	Palatine tonsil	27			
	Base of tongue	11			
Hurtuk *et al,* 2011	Larynx	4	16,3 ± 7,5	100	97
[42]	Retromolar trigone	1			
	Parapharynx	1			
	Total	44			
	Tonsil	7			
	Base of tongue	3			
Genden *et al,* 2009	Parapharynx	2	5,1	100	100
[16]	Hypopharynx	4			
	Palate	2			
	Total	18			
	Oropharynx	18			
Hans *et al,* 2011	Larynx	5	20,6	100	100
[49]	Hypopharynx	2			
	Total	25			
	Oropharynx	44			
Vergez *et al,* 2012	Hypopharynx	64	nd	98,4	nd
[50]	Larynx	18			
	Total	126			
	Pharynx	2			
Remacle *et al,* 2012	Supraglottic	2	9	100	100
[51]	Total	4			
	Tonsil	19			
Moore *et al,* 2009	Base of tongue	26	1–16	97,8	91,1
[41]	Total	45			
	Supraglottic	10			
Lawson *et al,* 2011	Pharynx	10	17	nd	91,7
[19]	Oral cavity	4			
	Total	24			
	Aryepiglottic fold	3			
	Piriform sinus	2			
	Pharynx	5			
Aubry *et al,* 2011	Base of tongue	3	6,5	100	100
[53]	Vallecula	1			
	Epiglottis	2			
	Arytenoid	2			
	Total	17			
Weinstein *et al,* 2010 [43]	Oropharynx	47	18–44	82	79
	Supraglottic 2	2			
Benazzo *et al,* 2012	Oropharynx	4	5,8	100	100
[54]	Total	6			
	Oral cavity	2			
White *et al,* 2010	Oropharynx	77	26	100	86.5
[45]	Larynx	10			
	Total	89			

**Table 3.** TORS operating room setup and surgical times.

Author	Primary site	Number of patients	Set-up time (min) Range (average)	Operative time (min) Range (average)
	Tonsil	7		
	Base of tongue	3		
Genden *et al,* 2009	Parapharynx	2	20–140 (54,6)	45–150 (84,0)
[16]	Hypopharynx	4		
	Palate	2		
	Total	18		
	Oropharynx	18		
Hans *et al,* 2011	Larynx	5	15–100 (25,0)	20–150 (70,0)
[49]	Hypopharynx	2		
	Total	25		
	Oropharynx	44		
Vergez *et al,* 2012	Hypopharynx	64	52 ± 46	90 ± 92
[50]	Larynx	18		
	Total	126		
	Pharynx	2		
Remacle *et al,* 2012	Supraglottic	2	10–60 (30,0)	60–125 (94,0)
[51]	Total	4		
Weinstein *et al,* 2007 [7]	Oropharynx	27	2–22 (9,0)	26–233 (84,0)
Park *et al,* 2009 [52]	Tonsil	5	15–25 (19,0)	40–50 (44,0)
	Tonsil	19	54–59 (68,6) first 10	45–320 (71,8) first 10
Moore *et al,* 2009 [41]	Base of tongue	26	14–28 (22,3) other 35	6–309 (71,3) other 35
	Total	45	14–59 (32,6) total	6–320 (71,4) total
	Supraglottic	10		
Lawson *et al,* 2011 [19]	Pharynx	10	10–60 (24,0)	12–180 (67,0)
	Oral cavity	4		
	Total	24		
	Aryepiglottic fold	3		
	Piriform sinus	2		
	Pharynx	5		
Aubry *et al,* 2011	Base of tongue	3	10–50 (20,5)	10–90 (39,7)
[53]	Vallecula	1		
	Epiglottis	2		
	Arytenoid	2		
	Total	17		
	Supraglottic 2	2		
Benazzo *et al,* 2012	Oropharynx	4	nd	35–135 (70,8)
[54]	Total	6		
